# Towards the simplification of MHC typing protocols: targeting classical MHC class II genes in a passerine, the pied flycatcher *Ficedula hypoleuca*

**DOI:** 10.1186/1756-0500-3-236

**Published:** 2010-09-05

**Authors:** David Canal, Miguel Alcaide, Jarl A Anmarkrud, Jaime Potti

**Affiliations:** 1 Estación Biológica de Doñana - CSIC, Department of Evolutionary Ecology, Av. Américo Vespucio s/n, 41092 Seville, Spain; 2 Department of Organismic and Evolutionary Biology, Harvard University, Cambridge, MA 02138, USA; 3 National Centre for Biosystematics, Natural History Museum, University of Oslo. P.O. Box 1172 Blindern, NO-0318 Oslo, Norway

## Abstract

**Background:**

Major Histocompatibility Complex (MHC) has drawn the attention of evolutionary biologists due to its importance in crucial biological processes, such as sexual selection and immune response in jawed vertebrates. However, the characterization of classical MHC genes subjected to the effects of natural selection still remains elusive in many vertebrate groups. Here, we have tested the suitability of flanking intron sequences to guide the selective exploration of classical MHC genes driving the co-evolutionary dynamics between pathogens and their passerine (Aves, Order Passeriformes) hosts.

**Findings:**

Intronic sequences flanking the usually polymorphic exon 2 were isolated from different species using primers sitting on conserved coding regions of MHC class II genes (β chain). Taking the pied flycatcher *Ficedula hypoleuca* as an example, we demonstrate that careful primer design can evade non-classical MHC gene and pseudogene amplification. At least four polymorphic and expressed loci were co-replicated using a single pair of primers in five non-related individuals (N = 28 alleles). The cross-amplification and preliminary inspection of similar MHC fragments in eight unrelated songbird taxa suggests that similar approaches can also be applied to other species.

**Conclusions:**

Intron sequences flanking the usually polymorphic exon 2 may assist the specific investigation of classical MHC class II B genes in species characterized by extensive gene duplication and pseudogenization. Importantly, the evasion of non-classical MHC genes with a more specific function and non-functional pseudogenes may accelerate data collection and diminish lab costs. Comprehensive knowledge of gene structure, polymorphism and expression profiles may be useful not only for the selective examination of evolutionarily relevant genes but also to restrict chimera formation by minimizing the number of co-amplifying loci.

## Background

For the last two decades, the Major Histocompatibility Complex (MHC) has drawn the attention of evolutionary biologists due to its importance in crucial biological processes, such as sexual selection and immune response in jawed vertebrates (reviewed in [1-3]). Classical MHC genes, unlike those classified as non-classical, usually display extensive levels of genetic variability and ubiquitous expression patterns [4]. Among classical MHC loci, most research has focused on the second and third exons of class I genes and the second exon of class II B genes because of their traditional consideration as primary targets of pathogen-mediated selection. These highly polymorphic exons encode the extracellular domains that bind and present foreign peptides (antigens) to specialised CD4+ and CD8+ lymphocytes. Subsequently, the recognition of the complex MHC molecule-foreign antigen by T-lymphocytes triggers adaptive immunity [5].

The characterization of classical MHC genes subjected to the effects of natural selection still remains elusive in many vertebrate groups [6]. MHC genes belong to an extremely dynamic multigene family characterized by frequent gene duplication and loss, presence of pseudogenes, gene conversion and chromosome reorganization [7-11]. Such complex evolutionary patterns could account for the substantial variation reported in MHC architecture and genome organization between and within different vertebrate groups [12,11,14], and sometimes even within the same species [15,16]. Like other multigene families, the MHC is thought to be the subject of both birth-and-death and concerted evolution, yet the distinction between the two evolutionary models is sometimes difficult and controversial [17]. The birth-and-death model implies the creation of new genes by gene duplication, some of them being functionally retained in the genome for long time periods whereas others become inactivated (pseudogenes) or deleted from the genome. The concerted evolution hypothesis predicts that MHC genes evolve as a unit, mainly because of repeated gene conversion events across different members of the gene family [17]. The implications of different forms of multigene family evolution are nevertheless crucial for MHC genotyping.

A prominent role of the birth-and-death evolutionary model has been typically associated with the mammalian MHC. Due to the independent evolution of MHC genes during long periods, MHC alleles usually form clusters according to loci. Such clusters allow tracing of orthologous relationships within and between different mammalian lineages [18,19]. This phenomenon has indeed facilitated the design of locus-specific primers across different mammalian groups (e.g. [20-22]). Non-mammalian lineages, on the other hand, usually exhibit a lack of orthologous relationships even on short evolutionary time scales [7,19]. In those groups, MHC sequences commonly fail to cluster according to loci [23-25], and, consequently, the assignment of alleles to particular genes becomes challenging. This phenomenon has been mainly attributed to concerted evolution that manifests in the homogenization of DNA sequences among different loci [26,17]. Therefore, high rates of concerted evolution hinder MHC typing protocols due to co-amplification of multiple loci (e.g. [27,23]) and increased risk of chimera formation during PCR amplification [28]. Taxa exhibiting extraordinarily high numbers of gene duplications and pseudogenes, such as songbirds and some fish, may be especially problematic [29-33].

Degenerate primers targeting conserved coding regions of exon 2 have proven successful for the isolation of MHC class II B sequences in non-model avian species [34,35,27,23]. Particularly in passerines, degenerate primers are expected to target (multiple) classical MHC genes, non-classical MHC genes and even pseudogenes (e.g. [36,32,33]). In this respect, a focus on evolutionarily relevant loci is needed in these species to diminish both laboratory efforts and costs. Despite strong evidence of concerted evolution in the avian MHC [37,23,31], a few studies in birds have demonstrated that comprehensive knowledge of gene structure can be critical for the design of locus-specific primers that amplify the entire coding sequence of the targeted exon 2 [10,38-41]. In this study, we have applied a multi-step PCR approach to obtain genomic MHC sequences in passerines (including both introns and exons). Our main goal was to test the suitability of flanking intron sequences to assist the specific amplification of the entire coding sequence of exon 2 from classical MHC class II B genes in passerines, a particularly challenging group regarding MHC genes.

## Methods

### Study Species

We used the pied flycatcher *Ficedula hypoleuca (Aves: Muscicapidae) *as a model species. We also used eight non-related species to get a preliminary glimpse about the suitability of our molecular approach across other songbird families. The selected species were the white wagtail *Motacilla alba (Motacillidae)*, the common raven *Corvus corax (Corvidae)*, the European robin *Erithacus rubecula (Muscicapidae)*, the woodchat shrike *Lanius senator (Laniidae)*, Dupont's lark *Chersophilus duponti (Alaudidae)*, the Sardinian warbler *Sylvia melanocephala (Sylviidae)*, the trumpeter finch *Bucanetes githagineus (Fringillidae) *and the chiffchaff *Phylloscopus collibita (Phylloscopidae)*.

### DNA and RNA extraction

Genomic DNA from five unrelated pied flycatchers was extracted from blood samples using the E.Z.N.A Blood extraction kit (Omega Bio-Tek, GA, USA). We used the HotSHOT protocol [42] to obtain genomic DNA from ethanol-preserved blood samples from one specimen of each additional passerine species mentioned above. To discern between expressed MHC genes and non-functional pseudogenes, total RNA was isolated from approximately 100 μl of fresh blood taken from one pied flycatcher individual using TRIzol^® ^LS Reagent (Invitrogen, CA, USA) according to the supplier's protocol. About 1 μg of total RNA was treated with DNase I (Sigma-Aldrich, MO, USA) before being reverse transcribed with the iScript™ cDNA synthesis kit (BioRad, CA, USA) to control for the possible amplification of target loci from genomic DNA. The cDNA was subsequently used as template for PCR amplification (see below).

### PCR Amplification of genomic MHC fragments in passerines

**Figure 1:**
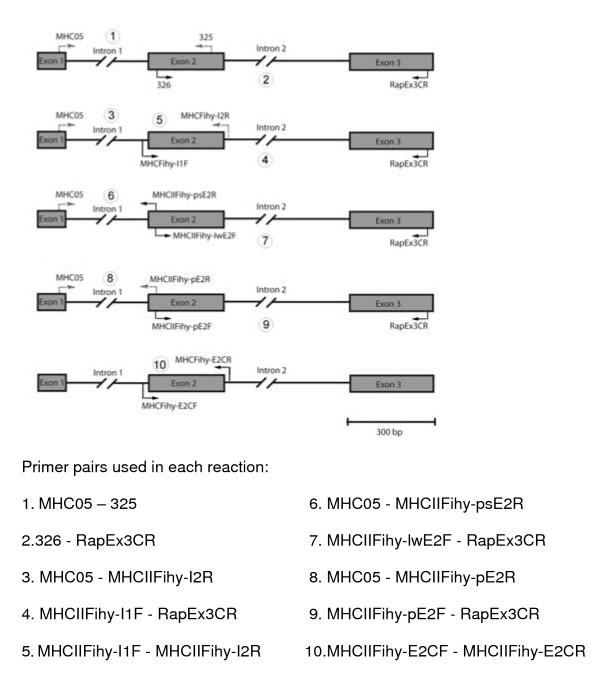
**Reactions carried out in this study to amplify MHC class II B sequences from genomic DNA in passerines**.

For each passerine species, we used two sets of primers targeting conserved regions of MHC class II B genes in birds: MHC05 [10] and 325 [27] amplified genomic fragments spanning exons 1 to 2, whereas using primers 326 [27] and RapEx3CR [23] a partial region of exon 2, the entire intron 2 and a stretch of exon 3 were amplified (Table 1 and Figure 1; steps 1 and 2). The logic behind this step was to examine the intron sequences flanking exon 2 among species and among loci. PCRs were carried out using a PTC-100 Programmable Thermal Controller (MJ Research) in a final volume of 30 μl containing 1 unit of a commercial Taq Polymerase (Bioline, London, UK), 1× manufacturer-supplied buffer (Bioline), 2.5 mM MgCl_2_, 0.25 mM of each dNTP, 5% Dimethyl sulfoxide (DMSO), 10 μg of BSA (Bovine Serum Albumin - Amersham Biosciences, Uppsala, Sweden), 10 pmoles of each primer and 1 μl of DNA extracts. PCRs were performed according to a touch down protocol from 66°C to 50°C (N = 16 cycles) plus 19 cycles of annealing temperatures at 50°C. Cycling programs consisted of a first denaturing cycle of 3 min at 94°C, plus subsequent steps of 94°C for 40 s, annealing steps for 40 s and extension steps at 72°C for 40 s. PCR amplicons were cloned and sequenced as described below except in the case of the pied flycatcher (see the next subsection for a detailed description of the methods used in this species).

**Table 1:** List of primer sequences used and/or developed in this study for PCR and sequencing. Standard IUB codes are used for degenerate primers.

Primer Name	Sequence (5'-3')	reference
326	GAGTGYCAYTAYYTNAAYGGYAC	Ekblom et al. (2003)
325	GTAGTTGTGNCKGCAGTANSTGTCCAC	Ekblom et al. (2003)
MHC05	CGTRCTGGTGGCACTGGTGGYGCT	Miller and Lambert (2004)
RapEx3CR	CAGGCTGRCGTGCTCCAC	Alcaide et al. (2007)
MHC-F1	GAGTGTYVCTTCATTAACGGCAC	Anmarkrud et al. 2010
MHC-R1	CKCGTAGTTGTGCCGGCA	Anmarkrud et al. 2010
MHCIIFihy-I1F	CCTGYACAAACAGRGKTKTTCC	This study
MHCIIFihy-I2R	GCTCTGCCCCACGCTCAC	This study
MHCIIFihy-pE2R	ACCTCACCTTCTCCGTGC	This study
MHCIIFihy-pE2F	AAYGGCACGGAGAAGGTG	This study
MHCIIFihy-lwE2F	CATTAAYGGCACCAGCCGG	This study
MHCIIFihy-psE2R	TCCTCTCCACCAACCTCACGCA	This study
MHCIIFihy-E2CF	CCGTGTCCTGCACACACAGC	This study
MHCIIFihy-E2CR	GGGACASGCTCTGCCCCG	This study
MHCIIPas-E2iF	GAGTGTYACTTCATTAACGGCAC	This study
MHCIIPas-E2iR	CYNGTAGTTGTGNCGGCAG	This study

### Targeting classical MHC class II B genes in pied flycatchers

Genomic fragments spanning exons 1 to 2 and exons 2 to 3 (reactions 1 and 2, Figure 1) were directly sequenced (see methods below) using primers 325 and 326, respectively, in the case of the five pied flycatchers. Although direct sequencing chromatograms were mostly noisy, we were able to design new primers in conserved intron 1-exon 2 (MHCIIFihy-I1F) and exon 2-intron 2 junctions (MHCIIFihy-I2R, Table 1 and Figure 1). These nucleotide positions were among those of best quality across sequencing chromatograms and we failed to detect nucleotide polymorphisms within or between individuals. Primers MHCIIFihy-I1F and MHCIIFihy-I2R were used in combination with primers MHC05 and RapEx3CR to generate a pool of genomic fragments along the MHC class II domain (steps 3, 4 and 5, Figure 1).

Primers MHC05 and MHCIIFihy-I2R (reaction 3, Figure 1) preferentially amplified an oligomorphic gene in the five individuals. Molecular cloning and sequencing (see below) revealed the occurrence of six different alleles and four non-synonymous nucleotide substitutions in exon 2 (GenBank Acc No. GU390299-GU390301). However, the examination of cDNA sequences (see below) confirmed that this locus was transcribed and may therefore represent a non-classical MHC gene. Primers MHCIIFihy-I1F and RapEx3CR (reaction 4, Figure 1), on the other hand, targeted at least one pseudogene as suggested by the occurrence of stop codons and frame shift mutations in the coding region of exon 2 (GenBank Acc. No. GU390297). Finally, direct sequencing of the PCR products obtained with the new primers MHCIIFihy-I1F and MHCIIFihy-I2R (reaction 5, Figure 1) denoted the co-amplification of non-classical MHC genes and pseudogenes along with highly polymorphic, classical MHC genes. We realized about the amplification of polymorphic MHC genes after comparing the ambiguous nucleotide positions among the direct sequencing chromatograms obtained from different individuals.

**Figure 2:**
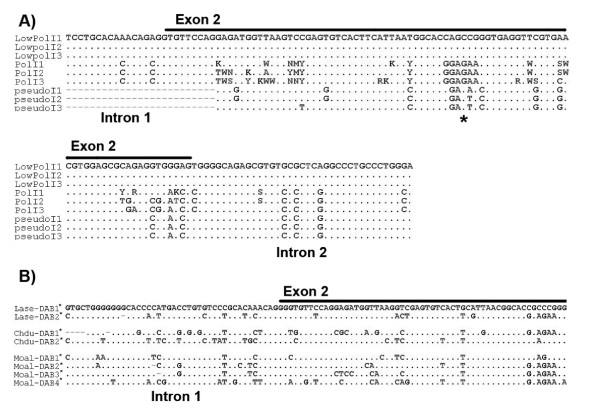
**A. Nucleotide differences among low polymorphic, high polymorphic MHC class II genes and pseudogenes in the pied flycatcher**. Sequences obtained from three different individuals are shown. Motifs allowing the design of loci-specific primers within exon 2 are pointed out with an asterisk. **B**. Nucleotide variations in the flanking introns of two presumably distinct MHC class II B genes of the woodchat shrike (Lase) and Dupont's Lark (Chde) along with four putatively distinct genes isolated in the white wagtail (Moal). Genes are labelled as DAB1*, DAB2* and so on. Asterisks indicate that the identity of these putative genes is not still confirmed, and future studies may confirm their identity.

In the following step, we tried to obtain intron sequences flanking exon 2 for each type of MHC loci (i.e. non-classical, classical and pseudogenes) using direct sequencing. To this aim, we profited from short nucleotide motifs within the sequence of exon 2 differing among loci (Figure 2A). This information was used to design primers for selective amplification and sequencing of specific intron sequences. Thus, primers MHCIIFihy-lwE2F (Table 1 and reaction 7, Figure 1) and RapEx3CR amplified intron 2 of non-classical MHC genes. Primers MHCIIFihy-pE2R and MHCIIFihy-pE2F (Table 1), in conjunction with primers MHC05 and RapEx3CR, amplified intron 1 and intron 2 sequences of classical MHC genes (reactions 8-9, Figure 1). We nonetheless failed to amplify intron 1 sequences from pseudogenes with primers MHCIIFihy-psE2R and MHC05 (Table 1 and reaction 6, Figure 1). This may result from the lack of this region in pseudogenes or due to the presence of extremely long introns difficult to amplify with our PCR protocol.

The alignment of flanking intron sequences showed differences in nucleotide composition suitable for the design of loci-specific primers (Figure 2A). Thus, in a last step we designed a set of new primers (MHCIIFihy-E2CF and MHCIIFihy-E2CR; Table 1 and reaction 10, Figure 1), aimed to specifically amplify classical MHC genes while overcoming the co-amplification of low polymorphic and pseudogenes. Direct sequencing revealed that highly polymorphic genes might share common flanking intron sequences (data not shown) and, therefore, the design of locus-specific primers was not feasible within this group.

### PCR amplification of cDNA

cDNA was amplified in one pied flycatcher individual employing the MHC class II exon 2 specific primers pair, MHC-F1 and MHC-R1 ([32], Table 1). The target of interest was amplified using the cDNA as template with similar PCR conditions and PCR purification approach as described in Anmarkrud et al. (2010; ref. [32])

### Molecular cloning and sequence analyses

PCR amplicons from genomic DNA and cDNA were cleaned-up in Microcon centrifuge tubes (Millipore) and subsequently cloned into bacterial plasmids using the PGEM-T easy vector system II (Promega, WI, USA). MHC inserts from positive clones were amplified as described above using the vector specific M13 primers. The PCR products were visualized on 1.5% agarose gels and inserts suspected to contain the target loci were sequenced using the BigDye 1.1 kit (Applied Biosystems, CA, USA). Between 8 and 16 positive clones per individual were analysed for PCR products obtained during the PCRs 1 to 4 (Figure 1). A total of 100 clones (20 per individual) were randomly screened for PCR products described in step 10 (Figure 1). Labelled fragments were resolved in an ABI3130xl automated sequencer (Applied Biosystems). MHC sequences were edited and aligned in BioEdit v. 7.0.9 [43]. The phylogenetic relationships among MHC sequences were visualized using Neighbor-net networks constructed in SplitsTree 4.0 [44] according to the Kimura-2-parameter model.

### Tests for selection

An excess of non-synonymous (d_N_) over synonymous (d_S_) substitutions characterizes coding sequences under positive selection [45]. Functional constraints in protein structure and function are translated, on the contrary, into an excess of synonymous substitutions (i.e. stabilizing or purifying selection). To detect selective signatures, d_N_/d_S _rates were calculated in MEGA 4.1 [46] using a Modified Nei-Gojobori method with Jukes-Cantor correction. Standard errors were calculated with 1,000 bootstrap replicates. Codons thought to be involved in antigen recognition were analysed independently from those presumably not involved in such function. We used information derived from the well-studied MHC class II molecule of humans [47] to delimitate putative antigen-binding regions. Statistical support for positive selection was evaluated through Z-tests run in MEGA 4.1.

## Results

### Genetic diversity and molecular evolution of classical MHC class II genes in pied flycatchers

**Figure 3:**
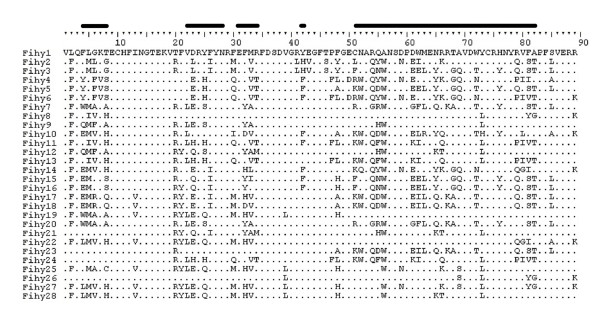
**Predicted amino acid sequences of 28 MHC class II alleles in the pied flycatcher**. Dots indicate identity with the top sequence. Black bars indicate the main coding regions exhibiting strong positive selection in the human MHC class II molecule (Brown et al. 1993).

**Figure 4:**
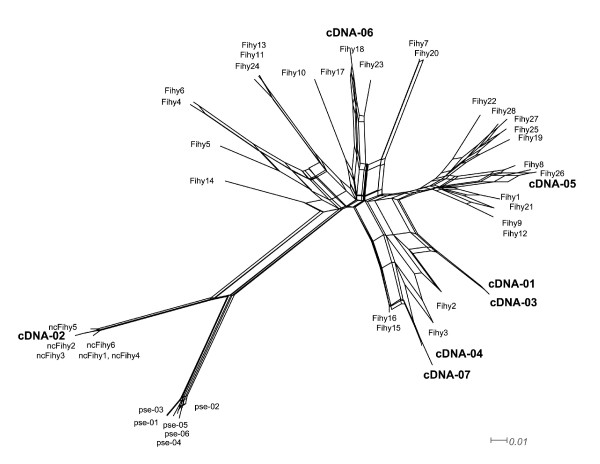
**Neighbor-net network of exon 2 sequences isolated from classical (Fihy1-28), non-classical (ncFihy1-6) and at least one MHC class II B pseudogene (pse01-06) in five pied flycatchers**. Seven cDNA sequences (cDNA01-07) isolated from a different individual are also shown.

We successfully and selectively amplified highly polymorphic, classical MHC class II genes in pied flycatchers using primers MHCIIFihy-E2CF and MHCIIFihy-E2CR (Table 1, step 10 in Figure 1). The analysis of 100 clones across the 5 individuals revealed 28 class II alleles translated into 28 amino acid sequences (GenBank Acc. No GU390232-GU390259, see Figure 3). For each individual, about 20% of the cloned alleles suspiciously resembled chimeric sequences or base misincorporations during bacterial replication and were discarded. In this respect, allele similarity was much higher within individuals than among individuals. Positive clones interchanging the first 30 bp of the 5' end of exon 2 were abundant. This finding hints at strong competition during the completion of PCR amplicons and the use of incomplete PCR amplicons as templates for subsequent amplification steps. The removal of putatively false and spurious alleles from our data set revealed between 5 and 8 alleles per individual, a finding in agreement with co-amplification of minimum 4 classical MHC class II B loci in pied flycatchers. The analysis of 267 bp of the exon 2 revealed high genetic polymorphism, with a large number of segregating sites (S = 112) resulting from 159 mutations, an average nucleotide diversity among sites (π = 0.167) and 44.63 nucleotide differences, on average, among alleles. For those codons located within putative antigen binding regions, non-synonymous substitutions were remarkably more frequent than synonymous substitutions (dN = 0.587 ± 0.084; dS = 0.190 ± 0.043; Z-test, P < 0.001). This was not the case for those codons not presumably interacting with antigens directly (dN = 0.054 ± 0.016; dS = 0.058 ± 0.021; Z-test, P = 0.79). Phylogenetic networks allow distinguishing a large cluster of sequences containing exon 2 sequences from classical MHC loci and a different cluster containing exon 2 sequences from non-classical MHC loci. Pseudogene sequences failed to intermingle with either of these two distinct clusters (Figure 4). A clustering of sequences according to loci is not evident within the exon 2 sequences derived from classical MHC genes. The phylogenetic network suggests the occurrence of divergent and recombining allele lineages that are shared among different loci instead. Finally, we isolated up to seven different cDNA sequences from the same individual. These cDNA sequences intermingle with the two clusters representing classical and non-clasical genes. Some of the cDNA sequences obtained in the individual investigated were identical or related to the sequences isolated from genomic DNA in other individuals (see Figure 4). Thus, our cDNA sequence data confirmed that both classical and non-classical MHC class II genes were transcribed in blood.

### Cross-amplification of genomic MHC fragments in passerines

**Figure 5:**
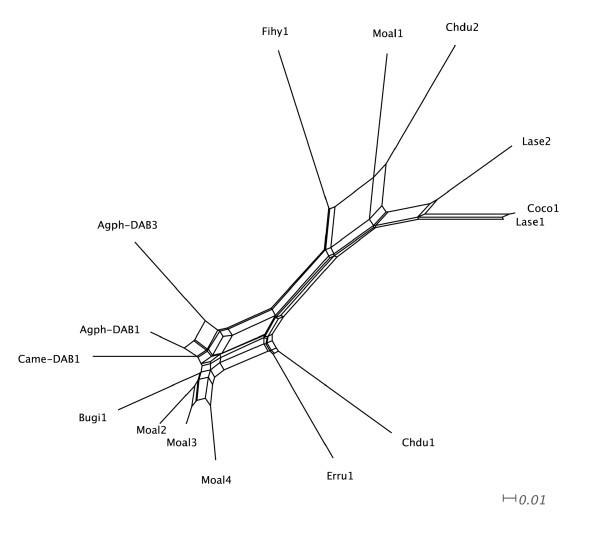
**Neighbor-Net network of complete intron 1 sequences isolated in this study plus those isolated in the red-winged blackbird *Agelaius phoeniceus *(AF030997.1) and the house finch *Carpodacus mexicanus *(AF205032.1)**. The sequences isolated from different species are labelled with different numbers.

Primers 326 and 325, in combination with MHC05 and RapEx3CR (equivalent to reaction 1 and 2, Figure 1) successfully amplified MHC class II genomic fragments across a wide variety of passerine species. Sequences from the white wagtail (GU390288-GU390293), common raven (GU390281-GU390283), European robin (GU390284), woodchat shrike (GU390281-GU390283), Dupont's lark (GU390277-GU390280), Sardinian warbler (GU390294-GU390296), trumpeter Finch (GU390273-GU390276) and chiffchaff (GU390293) were deposited in GenBank (see also Additional files 1 and 2). Our set of MHC sequences reported intron 1 sizes ranging from 299 to 478 bp and intron 2 sizes ranging from 190 to 350 bp in the species investigated. In those cases where we failed to obtain complete intron sequences from clones (especially in the case of intron 2), intron size was estimated through examination of 1.5% agarose gels. The alignment of intron sequences suggested the co-amplification of multiple copies in some species, such as the white wagtail, the woodchat shrike and Dupont's lark (Figure 2B). The phylogenetic network of intron 1 did not cluster according to species (Figure 5). This finding thus suggests that some regions of the multigene family can be gene conversion free and concerted evolution may not be ubiquitous throughout all the mutigene family. For the chiffchaff, the European robin, the common raven, the trumpeter Finch or the Sardinian warbler, our PCR experiments seemed to preferentially amplify particular MHC fragments (see Additional Files 1 and 2 for sequence data). However, the low number of clones analysed per species prevents this information being conclusive so far. All isolated sequences seemed to be putatively functional, as manifested by the lack of stop codons or frameshift mutations. However, not all the sequences obtained using both sets of primers were overlapping and we could not create contigs in all cases. As a result, more detailed examination for other species rather than the pied flycatcher is needed.

## Discussion

In this study we have for the first time isolated both coding and non-coding sequences corresponding to classical and non-classical MHC class II B genes in the pied flycatcher. The molecular protocol here described is also among the first ones demonstrating the utility of flanking intron sequences to simplify MHC genotyping in passerines. We show that intron sequences flanking the usually polymorphic exon 2 may assist the specific investigation of classical MHC class II B genes in species that, as passerines, are characterized by extensive gene duplication and pseudogenization [31]. Importantly, classical and highly polymorphic MHC genes are the primary targets of pathogen-mediated selection (reviewed by [2,3]) and the evasion of non-classical MHC genes with a more specific function and non-functional pseudogenes may accelerate data collection and diminish costs.

Our genetic data suggest the occurrence of at least 4 classical MHC class II B genes in pied flycatchers. However, suspicious evidence for chimera sequences makes this estimate far from conclusive. Additional studies more thoroughly minimizing PCR-mediated recombination (e.g. [28], see also discussion below) and even genetic inheritance analyses should add more light in this respect. We expect our primers to be related with a very low or non-existent incidence of null alleles. Primers are located immediately in the introns-exon 2 junction and some constraints in the mutation of this important region involved in the splicing of mRNA are therefore expected. Moreover, previous studies in birds of prey have shown that exon 2-introns boundaries of homologous genes are well conserved within related species and even when comparing different raptor lineages (e.g. [48,39]). The primers developed for the specific amplification of classical MHC genes in pied flycatchers also proved to cross-amplify MHC sequences in several passerine species of the Muscicapidae and Turdidae families (manuscript in prep.).

The cDNA sequences confirmed that both classical and non-classical MHC genes are expressed in pied flycatchers and are therefore functional. For classical MHC genes, we found an excess of non-synonymous substitutions, specifically for those amino acid positions that have been suggested to interact with antigens in the human MHC class II molecule ([47], see Figure 3). These regions have shown to accumulate positively selected sites in other avian lineages as well (e.g. [23,39,41]). All these findings corroborate the suitability of the classical MHC genes here described as relevant markers in eco-immunogenetics studies in the pied flycatcher.

### Concerted evolution and the simplification of MHC-typing protocols in passerines

Even though we successfully evade the co-amplification of pseudogenes and non-classical genes, our specific primers for classical MHC class II genes co-amplify multiple loci. Both exon 2 coding sequences (Figure 3) and flanking intron sequences (data not shown) suggest that concerted evolution may be responsible for the homogenisation of the genomic sequence of classical MHC class II genes in pied flycatchers and other passerines [31]. Concerted evolution is usually a considerable hindrance for the design of locus-specific primers (e.g. [23]) and this is the major reason behind our failure to design locus-specific primers in flycatchers. Under this scenario, sequencing the 3'-untransalted sequences (3' UTRs) of different genes has emerged as one of the very few alternatives to assign alleles to particular loci [10]. Detailed characterization of the MHC class II B in the barn owl *Tyto alba *has nevertheless shown that certain genomic regions are gene conversion free [40], a fact that allowed researchers to design locus-specific primers. In the case of the jungle fowl [38], single locus typing at both MHC class I and class II loci was possible due to the comprehensive knowledge of the MHC of the conspecific domestic chicken *Gallus gallus*. Similar strategies can now also be applied to the recently characterized MHC of the zebra finch *Taeniopygia guttata *[49] and have already proven useful in red-winged blackbirds *Agelaius phoeniceus *[50]. Neither of these scenarios is presently feasible in the case of the pied flycatcher, although next-generation sequencing technologies are expected to revolutionize the characterization of MHC complexes in the near future (Genome 10K Community of Scientists, 2009). An alternative to reduce the complexity of genotyping in those taxa with multiple classical genes may be the design of primers targeting only a subset of the allele repertoire, as explained in detail below.

### Risk of chimera formation during the co-amplification of multiple loci

Our genetic data demonstrate that PCR-mediated recombination is a serious source of false or spurious alleles when a large number of alleles are co-amplified simultaneously. The implications of this phenomenon are critical since many of the most popular MHC-typing protocols, including next-generation sequencing approaches [6], rely on PCR amplification at some stage. Reducing the number of cycles and extending elongation times during PCR amplification have been suggested to diminish the confounding effects of *in vitro *recombination [28] and genotyping strategies in passerines may therefore consider these precautions thoroughly. Taking a look at our alignments (Figs. 2 and 4), the design of primers targeting only a subset of the allele repertoire could be an adequate alternative to reduce the co-amplification of large numbers of alleles. Variability in MHC originates to a large extent by recombining alleles exchanging particular nucleotide motifs. This is evident, for instance, across the 5' end of the coding sequence of exon 2 in pied flycatchers (Figure 3). Despite implying more tedious sample manipulation in the lab, these approaches (further supported by non-denaturing capillary electrophoresis (SSCP or RSCA; reviewed in [6]) may be a useful alternative to minimize the incidence of false and spurious alleles. Regarding denaturing capillary electrophoresis, recent research [51] has shown that the simultaneous analysis of multiple fragments enhances our capabilities to discriminate between alleles when compared to the analysis of single PCR amplicons. Thus, partial digestion of PCR amplicons with restriction enzymes could be a promising strategy to improve resolution in cases similar to that documented here for the pied flycatcher. Alternatively, RSCA has also proven to be a very effective and high-throughput for the genotyping of duplicated MHC class II genes [52].

### Perspectives on additional passerine species

The cross-amplification of MHC sequences across a phylogenetically diverse array of passerine species decisively enhances the future applications of our molecular approach. Importantly, divergent introns-exon 2 boundaries within particular species such as the white wagtail, Dupont's lark or the woodchat shrine (Figure 2B) predict better opportunities for designing locus-specific primers or, at least, primers targeting a low number of loci. However, future studies in these and other species should tackle this issue in more depth to determine the broad utility of our protocol. We do believe that these data, although limited, may be really encouraging for the simplification of MHC-typing protocols in other passerine species since we have demonstrated that two single PCR reactions and the analysis of only a few clones are enough to isolate MHC sequences in the passerine species tested so far.

In some species, there was a trend for the preferential amplification of particular MHC fragments probably due to the large degeneracy of primers 326 and 325 (Table 1). For these reasons, we encourage the use of less degenerate primers (MHCIIPas-E2iF and MHCIIPas-E2iR) to minimize possible non-targeted products in passerines and biases towards the amplification of particular loci which may lead studies to miss important information on MHC structure. These primers were designed over conserved exon 2 motifs that emerged from an alignment of multiple passerine MHC class II sequences species. Nevertheless, these primers have not been tested in the present study and future studies will ascertain their utility. Finally, collection of genomic data will determine the suitability of similar approaches for MHC class I genes in passerines. The vast majority of studies so far have nonetheless dealt with expressed genes and genomic data are therefore scant in this avian lineage (e.g. [53,31,55]).

## Conclusions

This study highlights the advantages from the increasing knowledge in gene structure, polymorphism and expression profiles to simplify MHC typing protocols in passerine species. Importantly, the search for locus-specific primers opens the possibility to decisively overcome chimera formation and focus on computational inferences of gametic phase, one of the most promising alternatives for MHC genotyping in the future [6].

## Competing interests

The authors declare that they have no competing interests.

## Authors' contributions

DC, MC and JAA contributed equally to this work. They designed and carried out the experiments, performed the sequence alignment, the primer design and the data analysis, and drafted the manuscript. JP conceived the study, revised the manuscript and was responsible for the research grant that funded this study. All authors read and approved the final manuscript

## Supplementary Material

Additional file 1 **Intron I sequences obtained from different passerines, other than the pied flycatcher, in reaction 1 **(see Figure 1). Click here for file

Additional file 2 **Intron II sequences obtained from different passerines, other than the pied flycatcher, in reaction 2 **(see Figure 1). Click here for file
